# *Capra hircus* outliers markers in Brazil: Searching for genomic regions under the action of natural selection

**DOI:** 10.1590/1678-4685-GMB-2023-0084

**Published:** 2023-10-20

**Authors:** Francisco de A. Diniz, Jeane de O. Moura, Miklos M. Bajay, Leonardo Castelo Branco, Roosevelt D.S. Bezerra, Adriana M. de Araújo

**Affiliations:** 1 Universidade Federal do Piauí, Teresina, PI, Brazil.; 2 Instituto Federal do Piauí, Teresina, PI, Brazil.; 3 Universidade do Estado de Santa Catarina, Laguna, SC, Brazil.; 4 Universidade Federal Rural da Amazônia, Belém, PA, Brazil; 5 Embrapa Pantanal, Corumbá, MS, Brazil.

**Keywords:** Candidate genes, hardiness, outliers, non-neutral loci, tropical adaptation

## Abstract

Identifying genome regions subject to selection in local breeds of Brazil is an opportunity to elucidate the *C. hircus* genome plasticity, flowing the colonization history of the country. Using SNP Bead Chip Illumina 50K genotyping of Brazilian Anglo-Nubian (standardized breed) and Marota (local endangered population from the semiarid area of Brazil) to show outliers loci in genome regions subject to selection. After applying data quality control, 45,600 SNPs were included in this investigation. Principal component analysis (PCAdapt) and FDIST2 analysis signalized 14 genomic regions more affected by selection in the Brazilian dry zone environment. The genome study signalized regions that are close to the sequences of genes related to growth and embryonic skeletal development (FGF12, AMPD2, OSTN). In addition, flagged regions close to the genes UTSB2 and SLC5A2 contribute to research on functional adaptation with low water needs and poor nutritive diet to survive. Both genes encode proteins that act on osmotic pathways and avoid cell flooding on stress cell responses. Further studies on the genetic role and involvement of these outliers’ genomic regions, building a robust conceptual high-resolution map of natural selection drives, help to achieve hardiness candidate genes linked to the evolutionary history of *Capra hircus* in the semiarid area of Brazil.

## Introduction

*Capra hircus* was noticed as one of the first domesticated ruminants that occurred approximately 10,000 years ago (Zeder and Hesse, 2000). Flexible behavior and diet turn goats into one of the most important and older husbandry species, especially in arid mountains and areas of poor natural pastures. Furnishing suitable meat and milk production to humans, goats have significantly contributed to food security and so far alleviated the situation of poverty among farmers in developing countries (Lin et al., 2013; Periasamy et al., 2017).

Following the historic human migration and trade routes during Mercantilism, goats spread across the world, initially in Asia, Europe, and Africa, and from the Iberian Peninsula, reaching the Americas. This distribution was favored by genetic versatility and great adaptive capacity, such as resistance to different and adverse environmental conditions, expressing resistance to diseases and, mainly, thermal tolerance and nutritional adaptability (Aziz, 2010; Benjelloun et al., 2015; Amills et al., 2017).

In Brazil, goats began arriving in the 16th century from Portuguese expeditions. Brazilians’ local breeds have derived from the genetic flowing of surviving drive forces since then. Through governmental financial programs in the 1950s, modern African goat breeds such as nubians were introduced in Brazil aiming to improve the regional production of meat and milk and spread over the semi-arid area of the country. The harsh environmental conditions to which they were subjected, shaped genetically and phenotypically goats, creating enough variation to make Brazil the country with the greatest diversity of *C. hircus* in South America. There are 21 breeds, according to the Domestic Animal Diversity Information System (Mello de Araújo et al., 2009).

The F_ST_-heterozygosity outlier approach, proposed by Beaumont and Nichols (1996) is an indicator of genetic differentiation that allows the comparison of the population structure of different organisms. Loci or markers identified on the genome scan of genetic differential populations, which show higher or lower frequencies than expected under balanced conditions, are called outliers loci. Markers divergent or discrepant outliers have shown possibly under the action of natural selection, being favored or not by the environment (Beaumont, 2005; Pariset et al., 2009; Feng et al., 2015; Brito et al., 2017; Ahrens et al., 2018).

F_ST_ statistics is a widely used method, but a high number of false positive loci may occur, considering individuals grouped into populations (Excoffier et al., 2009; Bierne et al., 2013). An alternative is to combine approaches based on individuals, increasing the reliability of studies on selection signatures (Simianer et al., 2014; Brito et al., 2017). Methods based on multivariate analysis to genome scan individuals, such as PCAdapt are a plausible alternative to validate or identify selection signatures (Luu et al., 2017).

This study aimed to search for outliers, using an SNP 50K Bead Chip panel, employing different methodologies and associating regions of the *Capra hircus* genome with possible effects of the adaptation process, as well as identifying candidate genes that are under the action of natural selection.

## Material and Methods

### Ethics statement

All procedures performed with animals are following the rules of the Ethics and Research Committee of the Federal University of Piauí, registration number 058/14.

### Sampling data localization

The Mid-North is a vast transitional area among Brazilian biomes of the Cerrado savanna, Amazon, and semi-arid. The State of Piauí has borders with Maranhão, Ceará, and Pernambuco. The Piauí State spans an area of 251.529 km^2^ and has the third largest effective goat population in Brazil (available at Brazilian Statistic Institute of Geography https://sidra.ibge.gov.br/pesquisa/ppm/quadros/brasil/2021 ).

**Figure 1 -:**
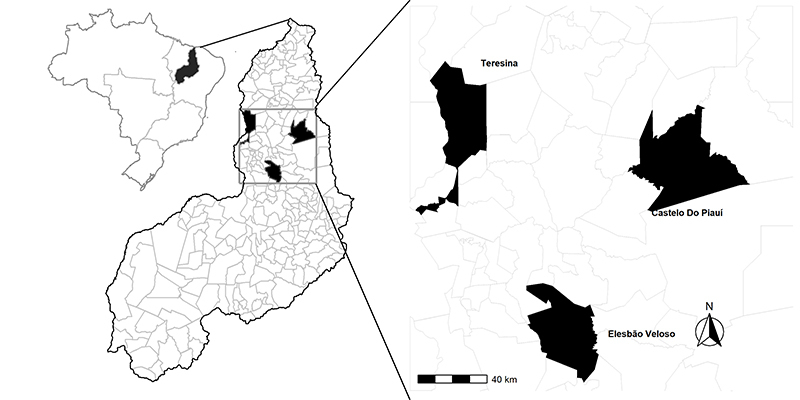
Geographic distribution of sampled goat breeds in Piauí - Brazil.

The data was of 96 mature live individuals in the Mid-North of Brazil. Two herds of goats - of different origins and at different times - were important for the semi-arid numerous flocks. Marota local rare breed (n = 86) and Anglo-Nubian breed (n = 10). The Marota local breed comes from the European colonialism history of South America. The samples in this study are from two herds: the National Conservation Program of Goats (latitude 05º19’20” south and longitude 41º33’09” west) and a private farm (latitude 06º12’07” south and longitude 42º08’25” west). The Anglo-Nubian standardized breed came to Brazil in the half of 1950. In the study, samples were from Piauí (05º02‟39.95” south, longitude 42º47‟03.70” west), aiming for comparative reference (Figure 1).

### Genotyping

Genotyping by 50K Illumina Goat Bead Chip, containing 53,347 SNPs evenly spaced in the chromosomes. The chip was manufactured using IlluminaTM Infinium technology, the iScan platform. The genotyping protocol was applied as established by the manufacturer (available at www.illumina.com) (Oliveira Moura et al., 2019a).

To evaluate the quality of the genotypic data, Plink (Purcell et al., 2007) was used and only the SNPs located on autosomal chromosomes were considered. Samples are filtered using a Call Rate of less than 80% and with a Minor Frequency Allele (MAF) <0.05 and which were in Hardy-Weinberg equilibrium P> 0.05 determined by Fisher’s exact test with 1000 permutations.

After first filtering, we applied quality control. Individual samples with a Call Rate <90%, that is, less than 90% of genotypes determined by the genotyping panel were excluded; heterozygosity above three standard deviations from the mean; identical genotypes (> 99.5%); and error in sex identification, in the case of individuals identified as males presenting heterozygous genotypes for markers on the X chromosome. The number of remaining SNPs, after quality control, was 45,600 (Anderson et al., 2010; Kijas et al., 2013).

### Determination of genomic regions under selection

Marota is a Brazilian rare local genetic resource from the semiarid region, which is in these days threatened with extinction (Mello De Araújo *et al*., 2009) due to the constant introduction of standardized genetic material in the region. The Marota *in situ* conservation lasts almost five decades due to government projects and private farm partnerships. In the institutional closed herds (called *In situ nucleus*), there is no migration or artificial directional selection. Due to the strategy used *in situ*, the genetic diversity within each subpopulation, natural selection is the main direct effect driving (Mello de Araújo et al., 2009). 

A common way of identifying *loci*, supposedly under selection in population genomics studies, is to investigate those that have high F_ST_ differentiation concerning their expected heterozygosity assuming the Hardy-Weinberg equilibrium (Beaumont and Nichols, 1996). Combining different and alternative approaches gives greater reliability to the studies of locating regions of the genome under the action of natural selection.

To detect marks potentially under selection, two available investigation methods were used. The hierarchical data analysis used the standard FDIST2 method based on the ARLEQUIN v 3.5.2.1 software (Excoffier and Lischer, 2010) and the fsthet R. package. Outliers marks were also identified in a principal component analysis (PCA) using the R package, called the PCAdapt R (Luu et al., 2017).

Standard FDIST2/fsthet method

The standard method FDIST2 (Beaumont and Nichols, 1996) was used, and implemented in the ARLEQUIN (Excoffier and Lischer, 2010) Appling methodology often uses the hierarchical model of islands. For each *locus*, allele frequencies were used to calculate the F_ST_ values conditioned to their heterozygosity in the HWE and to calculate the P values for each *locus* (Excoffier *et al*., 2009; Pariset et al., 2009).

To generate the joint distribution of F_ST_ versus heterozygosity we carried out 50,000 coalescent simulations, with 50 groups of 100 demes. Based on the 0.01 percentile, a 99% confidence interval limit for the expected distribution of the relation of the F_ST_ and heterozygosity. Flagged outliers loci was that showing atypical differentiation behavior (F_ST_) and located outside neutrality (Pariset et al., 2009).

### PCAdapt (version 2.0 of the R package)

PCAdapt R package identified the marks of outlier F_ST_, which performs genomic scans and detects genes under selection, using data based on individuals and multivariate analysis techniques, including principal component analysis (PCA). Initially, PCAdapt was executed to several main components K = 1, the number K explaining most of the variation, as recommended by the software authors (Luu et al., 2017), proceeding a statistical test to look for outlier SNPs and transformed into *p*-values to perform multiple hypothesis tests using Mahalanobis distances. 

Finally, the cut to detect outliers was chosen based on the *q*-value procedure implemented in the q-value R package (R Core Team 2020), using 0.01% as the rate limit of false discovery.

The distribution of p-values using a Quantil-Quantil plot of expected *-* values versus observed *p*-values and a histogram (Luu et al., 2017).

### False Discovery Rate (FDR)

The false-positive rate, conceptually, corresponds to the number of significant neutral *loci* (false positives) divided by the total number of neutral *loci* tested. Similarly, FDR is defined by the number of neutral false positives ratio by the total number of positive results (Lotterhos and Whitlock, 2014). The transformed results are used into *q*-values to correct multiple comparisons.

Forward, cutting *q* =0.01 value was used to define a positive result Among all loci showing *p*-values equal to or less than the observed locus, the *q-*value of a *locus* corresponds to the expected proportion of false positives. In this case, presupposed that *loci* that have *q* values of 0.01 must have an expected FDR of 1% (Luu et al., 2017).

### Genic content of regions identified as under selection

The genes in significant genomic regions were located according to the reference goat genome v1.0 (http://www.ncbi.nlm.nih.gov/genome?term=capra%20hircus). Gene searches for SNPs markers located in exon genes, using Ensembl Comparative Genomics Resources and the NCBI Gene database. In some cases, due to incomplete annotation of the goat’s genome, Ensembl’s BioMart tool (www.ensembl.org/biomart) was used to determine the orthologous cattle (*Bos Taurus*), sheep (*Ovis aries*), pigs (*Sus scroffa*) and (*Homo sapiens*) and others species with genetic IDs for each gene detected.

Searching the references and the QTL database of cattle, pigs, and sheep (available online at http://www.animalgenome.org/cgi-bin/QTLdb/index) to identify known phenotypes.

## Results

### Global F_ST_ value

The index of population differentiation FST is an important indicator to determine whether there is genetic differentiation between populations. The results showed that all paired FST values between populations were statistically significant (p<0.01). In our study, high genetic differentiation (FST=0.16) was observed between the Marota gene group and the Anglo-Nubian breed, indicating that Marota has considerable genetic diversity, suggesting that natural selection may have been a key factor in shaping the genetic diversity detected in this breed (Oliveira Moura et al., 2019a).

### Loci under selection and identification of genes under natural selection

**Figure 2 -:**
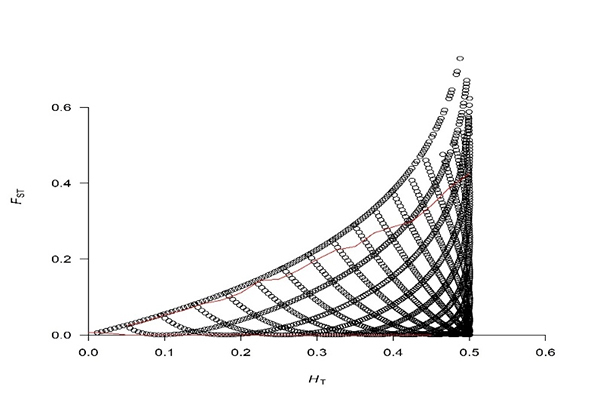
Scenario presented for the 1761 *outliers* marks that are suffering action of directional or disruptive natural selection, points above the red line.

The FDIST2 method, implemented in the Fsthet R software, detected a list of 1761 *loci outliers* that have an F_ST_ above the neutrality zone, with an average of 0.29 and an average heterozygosity of 0.35. These are located on all autosomal chromosomes, with chromosome 6 being the one with the highest number of *outliers*, as shown in Figure 2.

To detect genes under action of selection using a single test methodology may not present reliable and reproducible results. FDIST2 and PCA were implemented in this study (Simianer et al., 2014), according to Table 1.

**Table 1 -:** Genomic regions under selection and list of genes located in the region of the most significant SNPs: (A) detected by Arlequin’s FDIST2 and (B) detected by PCAdapt on correction (FDR=0.01).

Chr	Method	SNP outlier	Position	P-value	Genes
01	B	snp36295-scaffold4351695548*	74579244	0.044	FGF12, UTS2B, OSTN, CCDC50
03	B	snp6320-scaffold1223-315475*	12096695	0.086	Intergenic region
	B	snp25340-scaffold261 1087491*	31688141	0.088	DAB1, AMPD2, LOC102185621, LOC100861222
04	B	snp46417-scaffold641-1278697*	1278697	0.098	DNAJB6, UBE3C
06	B	snp59993-scaffold999-426450*	1736025	0.127	MARCH1, TMA16, NPY1R, NPY5R,TKTL2,
06	A	snp31742-scaffold354-420831*	420831	0.006	Intergenic region
	A	snp24457-scaffold248-1468420*	74829520	0.006	Intergenic region
07	B	snp41464-scaffold54493580*	68458023	0.158	Intergenic region
08	B	snp38279-scaffold4816-76898**	94632773	0.182	Intergenic region
	B	snp59201-scaffold972-291675*	95443929	0.182	Intergenic region
08	A	snp38279-scaffold4816-76898**	94632773	0.006	Intergenic region
	A	snp33341-scaffold391-3245653*	3665139	0.006	Intergenic region
	A	snp33316-scaffold391-2266280*	4644512	0.006	GALNTL6
09	B	snp36869-scaffold447-3340669**	80064765	0.204	Intergenic region
	B	snp8962-scaffold1324-311436*	89207416	0.206	Intergenic region
09	A	snp43771-scaffold588-1668067*	14724319	0.005	NKAIN2
	A	snp36869-scaffold447-3340669*	80064765	0.009	Intergenic region
11	B	snp18060-scaffold18510803188	62255976	0.238	PELI1
11	A	snp15213-scaffold1621-1365230*	58948520	0.006	LRRTM4
15	A	snp53215-scaffold801-192969*	9907114	0.007	Intergenic region
	A	snp41670-scaffold542-11277*	47407147	0.007	OTOG
16	B	snp52580-scaffold785-203051*	71615383	0.318	HHAT
21	A	snp18963-scaffold191-316399*	44518722	0.007	CFL2
22	B	snp21240-scaffold2073-421602*	25482065	0.400	Intergenic region
25	B	snp44135-scaffold6-477013**	27505380	0.445	SLC5A2, AHSP, ARMC5, LOC 102180368, LOC102181181
25	A	snp44135-scaffold6-477013**	27505380	0.005	SLC5A2, AHSP, ARMC5, LOC 102180368 LOC102181181
		snp57108-scaffold909-714756*	4282276	0.006	Intergenic region
26	A	snp55604-scaffold862-687700*	26426028	0.006	SORCS3

* p-value overlap on FDR correction

** p-value overlap at all approaches

**Figure 3 -:**
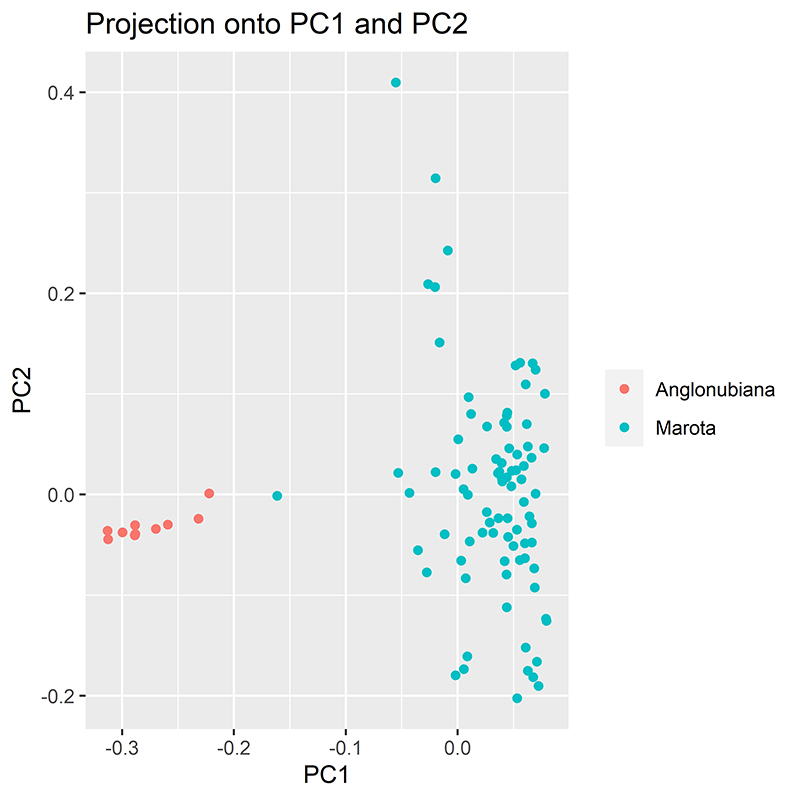
Dispersion in the Cartesian plane concerning the first two scores of the principal components.

Having as reference the origin of two populations of goats analyzed, the first main component (CP) genetically grouped the individuals. A histogram graphic of P values frequency distribution showed a frequency peak around p-value=zero, which is a strong statistic signal of the presence of loci outliers (Figure 3).

**Figure 4 -:**
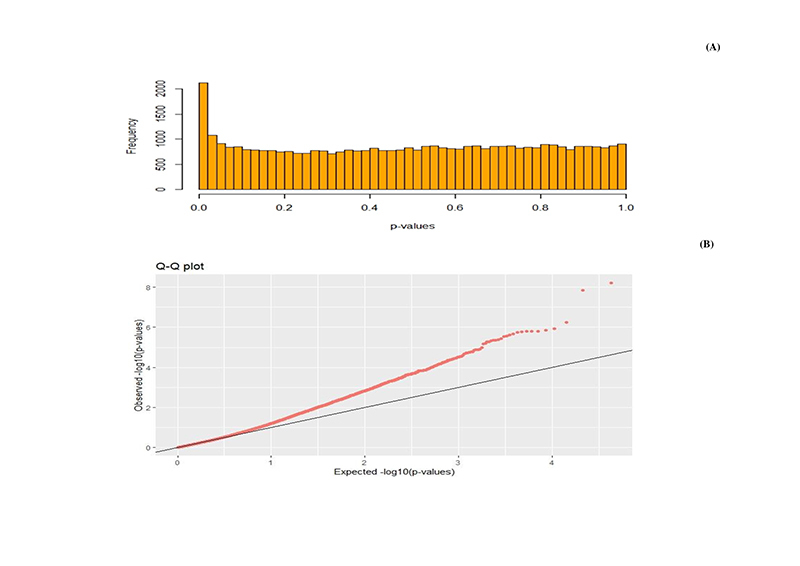
Distribution of P-values based on the histogram (A) and Distribution Q-Q plot (B).

The Quantile-Quantile plot of transformed p-value SNPs outlier markers have shown the fit of the frequency distribution of the data to a probability distribution (Settles et al., 2009). In this sense, the sample data set was used in PCAdapt R analyses to detect outliers SNPs. Then, the analysis of the Q-Q graph of the expected and observed *p*-values indicates that most of the p-values follow the expected uniform distribution, while *p*-values are lower than expected (FDR=0,01), therefore a condition of *outliers* marker confirmed. Fourteen SNPs were consistent outliers loci out of false discovery rate (FDR= 0.01) (Figure 4).

**Figure 5 -:**
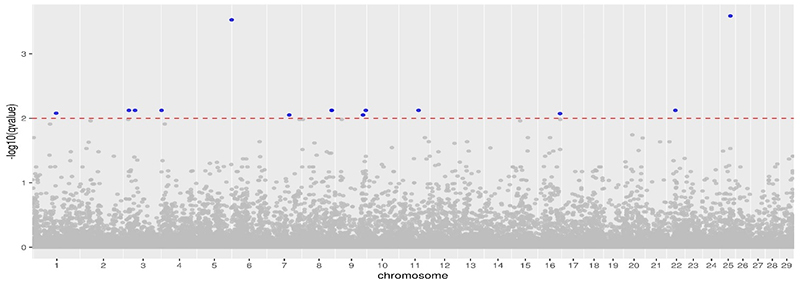
Scenario for the distribution of outliers with correction FDR=0.01 / PCAdapt.

Principal Components (PCAdapt) detected 1372 markers above the neutrality zone, corresponding marks discover on the genome scan, mostly on chromosome 27 and chromosome 06**.** Due to the large number of identified markers, we applied FDR q-value=0.01; in this case, 14 SNPs markers were detected as being under selection action, reducing the false discovering rate (Figure 5 and Table 1). 

### Mapping positively selected regions for genome annotations

Multiple outliers on different chromosomal regions were detected in the genome of Marota and Anglo-Nubian. Supposing that these regions contain non-neutral genes, they point to possible adaptive and/or productive biological pathways, such as meat/fiber production, body size, and hydrosaline balance control. These flagged regions of the genome are on different autosomal chromosomes (Table 1).

The identified genomic regions (Table 1) showed outlier markers located within gene sequences, which is a biological indication that those genes are non-neutral under the action of natural selection. Among the identified genes, we highlight OTOG, CFL2, LRRTM4, NKAIN2, GALNTL6, LOC102183030, HHAT, PELI1, DAB1, FGF2, DNAJB6, SORCS3. Genome close-flagged regions include UTS2B, OSTN, and SLC5A2, which are essentially related to the adaptation of goats in the hardness region.

## Discussion

Goat husbandry in Brazil has had different goals over time. The species gradually accumulated hardiness phenotypic traits, becoming adapted to tropical environments. Therefore, understanding the genetic and evolutionary mechanisms responsible for advantageous characteristics should be a guiding principle for the elaboration of conservation programs and animal genetic improvement. Furthermore, the development of complete genome sequencing technologies and the use of medium-density SNP chips allow the understanding of population genetic diversity (Benjelloun et al., 2015).

### Global FST analysis

The genetic diversity was higher between breeds than within individuals of standardized breeds, as expected in local breeds diversity (Grasso et al., 2014). Global F_ST_ of 0.16 shows the important genetic differentiation between Anglo-Nubian (an important standardized breed introduced in the 1950s in Brazil) and Marota (local goat population, no-directional selected), despite the years of coexistence in this region and the extensive breeding system in which the herds mate (Oliveira Moura et al., 2019a).

### Principal component analysis

For detecting outliers loci in the genome, the R package, PCAdapt has an additional advantage over the F_ST_ outlier method of the FDIST2, because it analyzes the population, without the need to subdivide it, that is, the data analysis is based on individuals (Debiec et al., 2013).

At first, genetic structuring analyses based on the main components revealed that the cluster number that explains the genomic variation data more clearly, between the Marota genetic group and the Anglo-Nubian breed, was K = 2 for analysis of SNPs markers. In this sense, when the subpopulations are represented in the Cartesian plane, constituted by the first two main components, there was a clear separation between the groups of animals (Figure2), with an individual from the distinct groups closely related genetically, which indicates that there is a mixed between breeds (Oliveira Moura et al., 2019a)

From the perspective of the distribution of the p-value in the histogram and in the quantile-quantile graph, the uniformity shown demonstrates calibration, as they are well distributed. However, the presence of *outliers* at the peak around zero is a strong indicator of loci under selection action (Moravčíková et al., 2018).

### Determination of genomic regions under selection

Table 1 shows several outliers loci, confirming previous SNP genome scan findings on goat genetic evolution to adaptive aspects in domestication and animal production processes (Brito et al., 2017; Bertolini et al., 2018). Table 1 set results of FDIST2 methodology- implemented in Arlequin- fsthet R, which identifies outlier marks with higher values of F_ST_ concerning heterozygosity; and PCAdapt, which detects genes under selection, using genomic data based on individuals and multivariate analysis techniques, including the principal component analysis (PCA). Regarding the different approaches for outliers detection, it was observed an overlap between markers in the chromosomal regions identified by the hierarchical island. Therefore, the model used was efficient to search goat genome marks under selection action during the evolutionary process of adaptation to the Tropical semiarid environment of South America, and even Africa.

There is an expectation that adaptation has molded the genetic diversity of native or naturalized groups of goats, in the semi-arid region, among them, highlighting the Brazilian local breed Marota. Identifying evidence of different spots of natural selection in a population, such as responses to the migratory process, natural selection, and genetic drift, can reveal important markers to comply with disadvantageous environmental conditions to produce animal livestock. Therefore, identifying biological functional *loci* involved in the local adaptive history of goats in Brazil could be the beginning point for understanding and characterizing their evolutionary genomic basis (Pariset et al., 2009; Hoban et al., 2016; Brito et al., 2017; Ahrens et al., 2018)

For this purpose, the recent use of SNPs markers was essential, which, due to their wide distribution in the different regions of the genome, can be located in coding regions or with regulatory functions. Therefore, SNPs provide opportunities to identify genetic variants under selection or genomic selection signatures. Thus, SNP allows for locating non-neutral behavioral *locus* or outlier between populations, as measured by the FST statistics and in different approaches (Hoban et al., 2016; Brito et al., 2017; Ahrens et al., 2018).

To understand the use of SNPs as markers to identify discrepant locus or outliers in the genome, it is worth mentioning that this marker is associated with other SNPs on the chromosome in which the mutation occurred. In this sense, the specific set of SNPs on a chromosome is called a haplotype. SNPs markers were close together in DNA exons regions and consequently, inherited together. When this marker is physically nearest to a locus (adaptive or not), if an allele in a haplotype is favored by natural selection, that allele will be more frequent in the population.

To avoid high rates of false marks, different approaches of PCA methods were investigated, which have the advantage of using related multivariate analysis techniques (Luu et al., 2017). Some analyses demonstrated that the decrease in the number of data samples has an insignificant effect on reliable positive ratios of identification when using multivariate approaches (Ahrens et al., 2018; Forester et al., 2018). PCA shows results expressed in simulations that involved only two populations. Therefore, use the hierarchical island model implemented in FDIST2 (Willing et al., 2012; Flanagan and Jones, 2017).

Both methodologies show overlaps of some markers distributed on the autosomal chromosomes of the sampled populations. Due to the evolutionary history of the Marota local breed, there may have been a population bottleneck or even population expansion, which could result in an excess of rare alleles (Oliveira Moura et al., 2019b). However, the consistency of peaks on chromosomes 06 and 25, were identified by both statistical approaches, showing strong evidence of *loci* under the action of selection, i.e., would hardly be characterized as false positives (Brito et al., 2017).

Chromosomes 01, 04, 06, and 08 showed several outliers flags spread over the genome. But it is well documented that the markers for several genes are not uniformly located by the genome and can be clustered in regions linked for productive and/or adaptive phenotypic traits, reported in other studies (Kim et al., 2016; Brito et al., 2017).

Previous research (Kim et al., 2016; Brito et al., 2017) signalized regions of the *C. hircus* genome as selection signatures in genes responsible to produce milk, meat, and coat fibers components. The present study, identified signatures of selection on the chromosome 06 gene region corroborates the information contained in another study (Brito *et al*., 2017). Particularly, the genome markers present in Marota local breed provide an opportunity to determine adaptive characteristics, correlated to the way of life of the goat animal in the semi-arid tropical regions of Brazil (Oliveira Moura et al., 2019a, b). 

### Description of the expected biological function of the outlier loci

The candidate genes on genomic regions associated with a functional understanding of specific biological and molecular processes and functions can appoint important outlier loci information. FGF12 (fibroblast growth factor 12), AMPD2 (adenosine monophosphate deaminase 2), OSTN (osteocrin) genes, express proteins that influence body size and skeletal and embryonic development (Willing et al., 2012; Bertolini et al., 2018). The SLC5A2 gene (solute carrier family 5 member 2) encodes protein that acts as a carrier for glucose and mineral salts in the kidneys (Kaji et al., 2018). The UTSB2 gene (urotensin 2), acts as a potent constrictor vessel (Debiec et al., 2013).

The small body size of the Marota breed (Mello De Araújo *et al*., 2009; Barros et al., 2011) and its correlation to the thermal regulation process could be explained (Benjelloun et al., 2015) for various domestic animals’ local populations in semi-arid regions. Pastures are generally of low nutritional value and temperatures are high, as these processes are controlled by genes under the action of natural selection, such as FGF2, OSTN genes (Brito et al., 2017; Kaji et al., 2018). Growth characteristics are genetically determined by natural selection toward reduced body size, in the opposite direction that any human effort to enhance growth or animal size by artificial selection in tropical arid and semi-arid environments. 

The SLC5A2 (solute carrier family 5 member 2) locus, which encodes the protein that acts as a carrier for glucose and mineral salts in the kidneys (Kaji et al., 2018), and the UTSB2 (urotensin 2) locus, which acts as a potent constrictor (Debiec et al., 2013), are highlighted. Most likely, these loci act by regulating the osmotic balance of goats in arid and semi-arid areas, where animals need to live with low amounts of water to consume. Different from the populations studied by other authors (Grasso et al., 2014; Brito et al., 2017), it is up to this work to discuss *loci outliers* related to adaptation to dry tropical environments.

In conclusion, SNPs outlier analysis allows robust genome maps of selection, identification of signals of candidate genes, and a better understanding of the evolutionary history of the species;

The PCA e FST approaches were efficient and complementary in elucidating the genomic regions under selection;

The evolutionary model allows highlighting important genes related to the semiarid environment adaptation of goats, flagging genes related to reduced body growth;

The water balance pathways and the reduced need for rich-in-water diets were traced to be relevant in the evolutionary history of goats in tropical regions.
